# Hydrogen sulfide production does not affect antibiotic resistance in *Pseudomonas aeruginosa*

**DOI:** 10.1128/aac.00075-24

**Published:** 2024-03-06

**Authors:** Lorenzo Caruso, Marta Mellini, Ortensia Catalano Gonzaga, Alessandra Astegno, Elena Forte, Adele Di Matteo, Alessandro Giuffrè, Paolo Visca, Francesco Imperi, Livia Leoni, Giordano Rampioni

**Affiliations:** 1 Department of Science, University Roma Tre, Rome, Italy; 2 Department of Biotechnology, University of Verona, Verona, Italy; 3 Department of Biochemical Sciences, Sapienza University of Rome, Rome, Italy; 4 CNR Institute of Molecular Biology and Pathology, Rome, Italy; 5 IRCCS Fondazione Santa Lucia, Rome, Italy; 6 NBFC, National Biodiversity Future Center, Palermo, Italy; Shionogi, Inc., Florham Park, New Jersey, USA

**Keywords:** antibiotic resistance, *Pseudomonas aeruginosa*, cystic fibrosis, H_2_S, antibiotic adjuvants

## Abstract

Hydrogen sulfide (H_2_S) has been proposed to protect bacteria from antibiotics, pointing to H_2_S-producing enzymes as possible targets for the development of antibiotic adjuvants. Here, MIC assays performed with *Pseudomonas aeruginosa* mutants producing altered H_2_S levels demonstrate that H_2_S does not affect antibiotic resistance in this bacterium. Moreover, correlation analyses in a large collection of *P. aeruginosa* cystic fibrosis isolates argue against the protective role of H_2_S from antibiotic activity during chronic lung infection.

## INTRODUCTION

Multi-drug resistant (MDR) bacterial pathogens rapidly spread, and only a few novel antibacterial drugs are in the pipeline (1). This alarming situation calls for new therapies to treat MDR infections (2), including the development of adjuvants that re-empower antibiotic action (3). In this context, hydrogen sulfide (H_2_S) production has attracted the attention of scientists (4, 5), as some studies demonstrated that endogenously produced H_2_S reduces bacterial susceptibility to antibiotics (6–9) and that H_2_S-producing enzymes are promising targets for the development of antibiotic adjuvants (10–13).

*Pseudomonas aeruginosa* is a primary opportunistic human pathogen, being responsible for over 300,000 deaths annually (14), and a major cause of chronic lung infection in individuals with cystic fibrosis (CF) (15). This bacterium possesses the genes coding for the H_2_S-synthesizing enzymes cystathionine γ-lyase (CSE), cystathionine β-synthase (CBS) (6, 10), and mercaptopyruvate sulfurtransferase (3MST) (7), although the role of 3MST in H_2_S production in *P. aeruginosa* remains unexplored. Additionally, *P. aeruginosa* utilizes sulfide:quinone oxidoreductases (SQR1 and SQR2) and the persulfide dioxygenase (PDO) to dispose of H_2_S and prevent sulfide accumulation (16). To date, the actual involvement of H_2_S production in *P. aeruginosa* resistance to clinically relevant antibiotics has been seldom investigated (6, 7, 10). Therefore, the potential efficacy of drugs targeting H_2_S production for anti-*P*. *aeruginosa* therapies remains uncertain.

**Fig 1:**
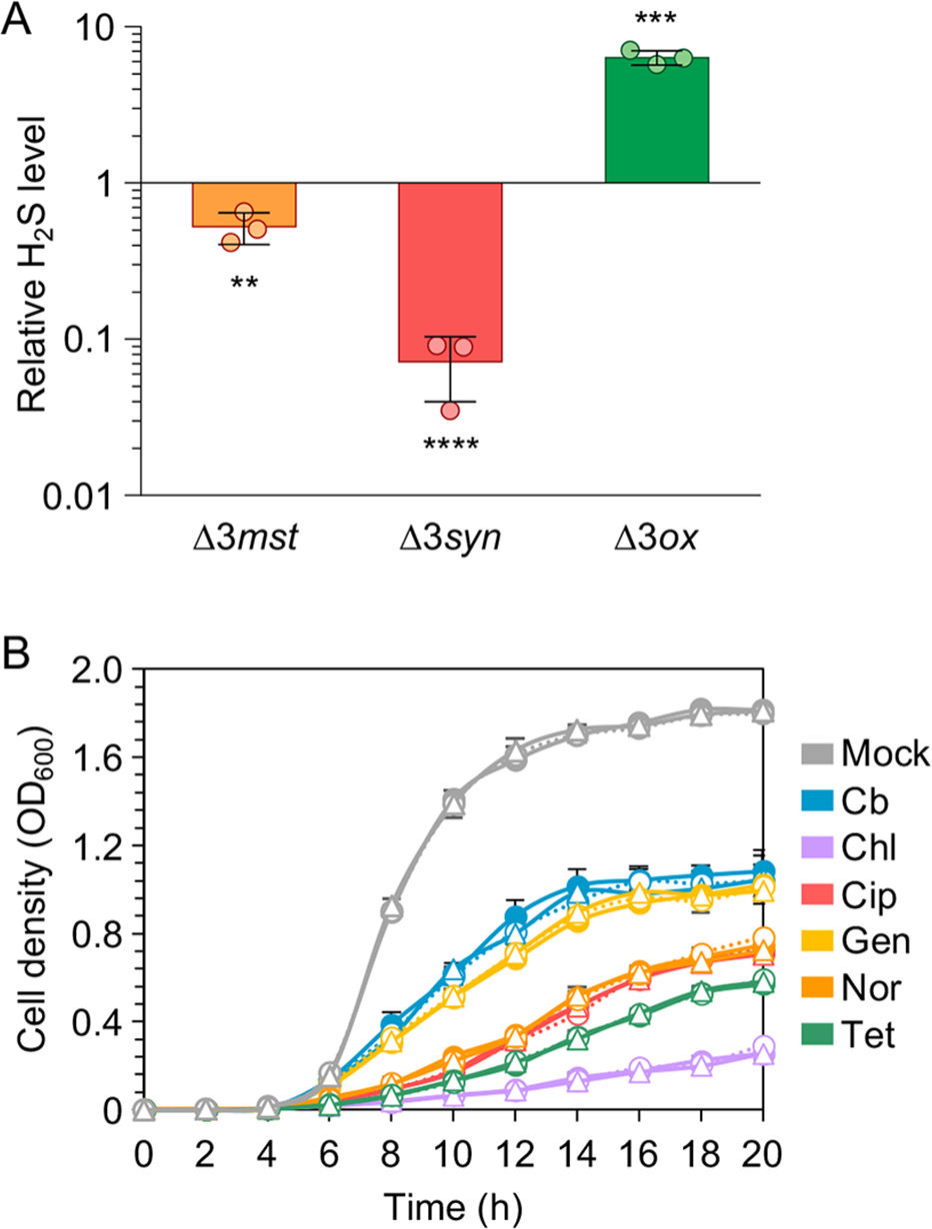
(**A**) Fold change in the H_2_S levels released by the ∆3*mst*, ∆3*syn*, and ∆3*ox* mutants relative to PAO1. H_2_S levels were determined via densitometric analyses of lead acetate-soaked paper strips exposed for 20 hours to the bacterial cultures grown in LB. The average values of three independent experiments, each performed on eight bacterial cultures, are reported with standard deviations. Asterisks denote statistically significant differences with respect to PAO1 (**P < 0.01; ***P < 0.001; and ****P < 0.0001; unpaired *t*-test). (**B**) Growth curves of PAO1 (solid lines, full circles), ∆3*syn* (dotted lines, empty circles), and ∆3*ox* (solid lines, empty triangles) in LB (mock; gray lines) or in LB supplemented with the indicated antibiotics at sub-MIC concentrations (1/4 MIC). Cb, carbenicillin (blue lines); Chl, chloramphenicol (purple lines); Cip, ciprofloxacin (red lines); Gen, gentamicin (yellow lines); Nor, norfloxacin (orange lines); and Tet, tetracycline (green lines). The average values of three independent experiments are reported with standard deviations.

To assess the effect of H_2_S production on *P. aeruginosa* antibiotic resistance, markerless deletion mutants producing higher or lower levels of H_2_S compared to the parental strain PAO1 were generated by multi-step allelic exchange (17, 18). H_2_S released by cultures of PAO1 and its isogenic mutants was quantified by using an optimized protocol based on lead acetate-soaked paper strips (6, 10, 16, 19), as detailed in Supplementary Materials and Methods. Briefly, this method relies on the staining of a lead acetate-soaked paper strip, whose intensity positively correlates with the amount of H_2_S released by the bacterial culture, as confirmed by using increasing concentrations of the H_2_S-donor sodium hydrosulfide (NaHS) (Fig. S1). This analysis revealed that 3MST contributes to H_2_S production in *P. aeruginosa*, as the ∆*3mst* mutant produced ca. 50% H_2_S relative to PAO1 in Lysogeny Broth (LB) (Fig. 1A). Based on this evidence, the ∆3*syn* mutant deleted in the three genes contributing to H_2_S production in *P. aeruginosa* (i.e., *3mst*, *cbs*, and *cse*) was constructed; this mutant showed <8% residual H_2_S levels compared to PAO1 (Fig. 1A). The same strategy was used to construct the ∆3*ox* mutant, deleted in the three genes implicated in H_2_S disposal in *P. aeruginosa* (i.e., *sqr1*, *sqr2*, and *pdo*); this mutant exhibited ca. 6.4-fold higher H_2_S levels compared to PAO1 (Fig. 1A).

The standard microdilution method (20) was used to perform MIC assays for the PAO1, Δ3*syn,* and Δ3*ox* strains grown in cation-adjusted Mueller-Hinton broth (MHB-II), LB, or tryptic soy broth supplemented with l-cysteine (TSB-cys). The addition of l-cysteine to the medium is known to increase H_2_S production (6). LB and TSB-cys were also used to reproduce experimental settings used in previous studies (6, 10). Antibiotics previously tested in studies focused on H_2_S production in *P. aeruginosa* (i.e., carbenicillin, chloramphenicol, ciprofloxacin, gentamicin, norfloxacin, and tetracycline) (6, 7, 10) and other antibiotics of clinical relevance for *P. aeruginosa* (i.e., colistin, meropenem, and tobramycin) were tested. As H_2_S is a volatile molecule, to limit its possible leakage, MIC assays in MHB-II were also performed by sealing the microtiter plates with an adhesive plastic sheet not permeable to H_2_S (Fig. S2). The Δ3*syn* and Δ3*ox* mutants produced lower and higher levels of H_2_S relative to PAO1, respectively, also in the experimental conditions used for the MIC assays (Fig. S3). MIC values of all the tested antibiotics were the same for PAO1, Δ3*syn,* and Δ3*ox* in all conditions (Table 1). Notably, the PAO1, Δ3*syn,* and Δ3*ox* strains showed comparable growth curves when treated with sub-MIC concentrations of previously tested antibiotics in LB (Fig. 1B) or in LB supplemented with l-cysteine or NaHS (Fig. S4). This demonstrates that H_2_S levels do not affect *P. aeruginosa* growth kinetics in the presence of antibiotics.

**TABLE 1:** MIC values for PAO1 and its isogenic mutants Δ3*syn* and Δ3*ox*

		MIC (µg/mL)^*a*^								
Medium	Strain	Cb	Chl	Cip	Col	Gen	Mer	Nor	Tet	Tob
MHB-II	PAO1	128	64	0.25	1	4	1	1	32	2
	Δ3*syn*	128	64	0.25	1	4	1	1	32	2
	Δ3*ox*	128	64	0.25	1	4	1	1	32	2
MHB-II wps	PAO1	128	64	0.25	1	4	1	1	32	2
	Δ3*syn*	128	64	0.25	1	4	1	1	32	2
	Δ3*ox*	128	64	0.25	1	4	1	1	32	2
LB	PAO1	128	64	0.25	1	4	1	1	16	2
	Δ3*syn*	128	64	0.25	1	4	1	1	16	2
	Δ3*ox*	128	64	0.25	1	4	1	1	16	2
TSB-cys	PAO1	128	64	0.125	1	4	1	0.5	32	1
	Δ3*syn*	128	64	0.125	1	4	1	0.5	32	1
	Δ3*ox*	128	64	0.125	1	4	1	0.5	32	1

^*a*^ Cb, carbenicillin; Chl, chloramphenicol; Cip, ciprofloxacin; Col, colistin; Gen, gentamicin; Mer, meropenem; Nor, norfloxacin; Tet, tetracycline; Tob, tobramycin; MHB-II wps, MHB-II sealed with plastic sheet; and TSB-cys, tryptic soy broth supplemented with 200 µM l-cysteine.

**Fig 2:**
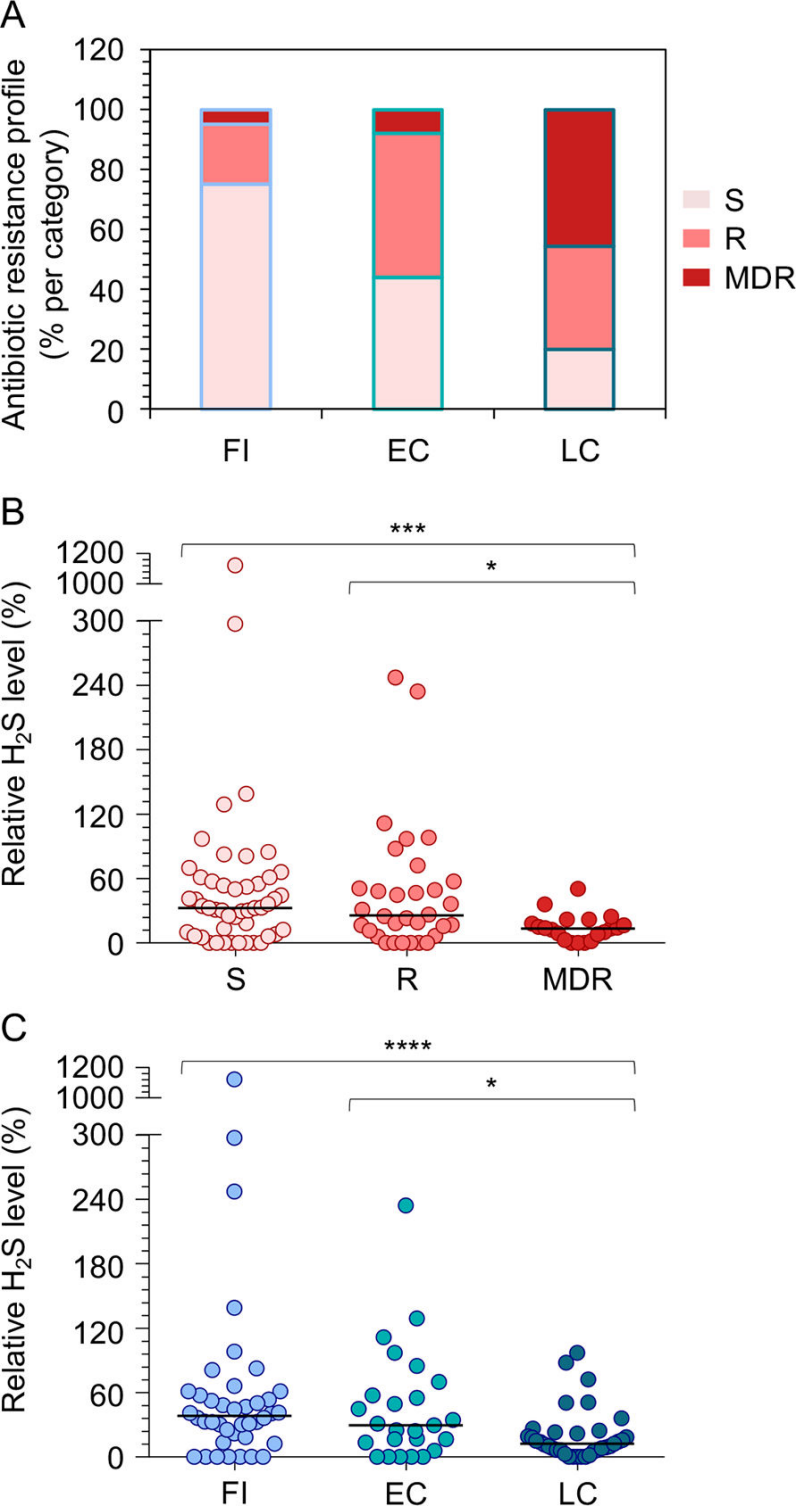
(**A**) Antibiotic resistance pattern of 100 CF isolates categorized by the duration of the lung infection (23, 24). FI, first isolate (*n* = 40); EC, early chronic (*n* = 25); LC, late chronic (*n* = 35); S, susceptible to all antibiotics tested (*n* = 48); R, resistant to one or two antibiotics of different classes (*n* = 32); and MDR, multi-drug resistant (non-susceptible to at least one agent in three or more classes of antibiotics) (*n* = 20). (**B**) Relative H_2_S levels released in LB by the CF isolates grouped by antibiotic resistance pattern. (**C**) Relative H_2_S levels produced in LB by the CF isolates grouped by the duration of the chronic lung infection. For panels **B** and **C**, each circle represents the average of three independent experiments for a given strain; the percentages refer to the H_2_S level measured in PAO1 in LB, considered as 100%. Horizontal lines indicate median values. Asterisks denote statistically significant differences between groups (*P < 0.05; ***P < 0.001; and ****P < 0.0001; Kolmogorov-Smirnov test).

Resistance to many clinically relevant classes of antibiotics is frequently observed in *P. aeruginosa* strains isolated from CF patients with chronic lung infection (21, 22). To assess a possible correlation between H_2_S production and antibiotic resistance in clinical isolates, we quantified the H_2_S levels produced by 100 clinical isolates of *P. aeruginosa* from CF lungs with distinct antibiotic resistance profiles (Fig. 2A) (23, 24). This analysis revealed that H_2_S levels are lower in resistant and MDR isolates relative to sensitive ones (Fig. 2B) and that a decrease in H_2_S production parallels the progression of chronic infection (Fig. 2C). Hence, high levels of H_2_S production appear to be counter-selected during *in vivo* infection in the CF lung, despite antibiotic resistance increases.

Overall, our data argue against the role of H_2_S in conferring antibiotic resistance during CF lung infection. While it is possible that specific host-associated stimuli could boost *P. aeruginosa* H_2_S production to protective levels *in vivo*, this possibility is discredited by the evidence that H_2_S does not protect *P. aeruginosa* from antibiotics even when produced at high levels, as observed in the ∆3*ox* mutant.

Our data do not support the protective role of H_2_S against antibiotics claimed for *P. aeruginosa* in previous studies (6, 7, 10). Similar discrepancies have been reported for *Staphylococcus aureus*; while some studies reported that H_2_S production confers antibiotic resistance to this bacterium (6, 10), a contrasting study indicated that endogenous H_2_S levels are not sufficient to protect *S. aureus* from aminoglycosides and that exogenous provision of H_2_S decreases *S. aureus* resistance to non-aminoglycoside antibiotics (25). H_2_S decreases antibiotic resistance in *Acinetobacter baumannii* too (26). Interestingly, a mutant of *Fusobacterium nucleatum* substantially deficient in H_2_S production gained significant sensitivity to nalidixic acid and resistance to kanamycin (9). By providing evidence that H_2_S production is not a defense mechanism against antibiotics in *P. aeruginosa,* our data support the notion that H_2_S does not act as a protective molecule in all bacterial species.

## SUPPLEMENTAL MATERIAL

Supplemental figures and tables and additional experimental details aac.00075-24-s0001.pdf Figure S1 (H_2_S calibration curve), Figure S2 (The plastic sheets used in this study are not permeable to H_2_S), Figure S3 (H_2_S production under conditions used for MIC assays), Figure S4 (Growth curves in the presence of antibiotics at sub-MIC concentration), Table S1 (Bacterial strains used in this study), Table S2 (Plasmids used in this study), Table S3 (Oligonucleotides used in this study), and detailed materials and methods.

ASM does not own the copyrights to Supplemental Material that may be linked to, or accessed through, an article. The authors have granted ASM a non-exclusive, world-wide license to publish the Supplemental Material files. Please contact the corresponding author directly for reuse.
