# Supplementary material for: Hydrogen sulfide production does not affect antibiotic resistance in Pseudomonas aeruginosa
Source: Antimicrob Agents Chemother. 2024 Mar 6;68(4):e00075-24. doi: 10.1128/aac.00075-24 (PMC10989007; doi:10.1128/aac.00075-24)
Supplement: Supplemental figures and tables and additional experimental details — Figure S1 (H2S calibration curve), Figure S2 (The plastic sheets used in this study are not permeable to H2S), Figure S3 (H2S production under conditions used for MIC assays), Figure S4 (Growth curves in the presence of antibiotics at sub-MIC concentration), Table S1 (Bacterial strains used in this study), Table S2 (Plasmids used in this study), Table S3 (Oligonucleotides used in this study), and detailed materials and methods. [file aac.00075-24-s0001.pdf]

Hydrogen sulfide production does not affect antibiotic resistance in *Pseudomonas aeruginosa*

Lorenzo Caruso[1*], Marta Mellini[1*], Ortensia Catalano Gonzaga[1§], Alessandra Astegno[2], Elena Forte[3], Adele Di Matteo[4], Alessandro Giuffrè[4], Paolo Visca[1,5,6], Francesco Imperi[1,5,6], Livia Leoni[1], Giordano Rampioni[1,5#]

[1] *Department of Science, University Roma Tre, Rome, Italy*

[2] *Department of Biotechnology, University of Verona, Verona, Italy*

[3] *Department of Biochemical Sciences, Sapienza University of Rome, Rome, Italy*

[4] *CNR Institute of Molecular Biology and Pathology, Rome, Italy*

[5] *IRCCS Fondazione Santa Lucia, Rome, Italy*

[6] *NBFC, National Biodiversity Future Center, Palermo, Italy*

SUPPLEMENTAL MATERIAL

Figure S1. H_2S calibration curve

Figure S2. The plastic sheets used in this study are not permeable to H_2S

Figure S3. H_2S production under conditions used for MIC assays

Figure S4. Growth curves in the presence of antibiotics at sub-MIC concentration

Table S1. Bacterial strains used in this study

Table S2. Plasmids used in this study

Table S3. Oligonucleotides used in this study

Detailed Materials and Methods

References

Figure S1

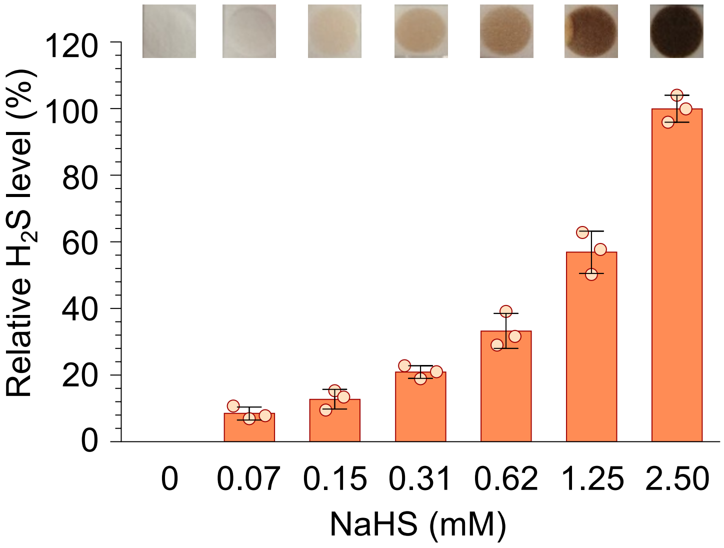

Figure S1. H_2S calibration curve

Relative densitometric analysis of lead acetate-soaked paper strips after 10-min reaction with H_2S released at increasing concentrations of NaHS in LB. The average values of three independent experiments, each performed on eight samples, are reported with standard deviations. The average value obtained with 2.5 mM NaHS was considered as 100%.

Figure S2

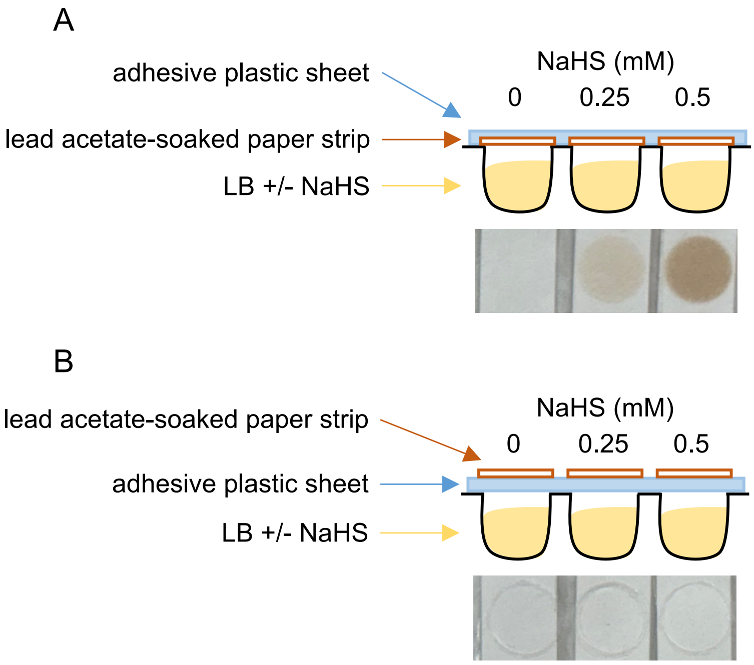

Figure S2. The plastic sheets used in this study are not permeable to H_2S

Schematic representation of the control experiment performed to test possible permeability to H_2S of the adhesive plastic sheets used to seal the 96-well microtiter plates (top panels), and the relative lead acetate-soaked paper strips after 10-min reaction with H_2S released at increasing concentrations of NaHS in LB (bottom panels). **(A)** The lead acetate-soaked paper strips were placed onto the microtiter plate, and the adhesive plastic sheet was placed on top of them to seal the microtiter plate. **(B)** The adhesive plastic sheet was used to seal the microtiter plate, and the lead acetate-soaked paper strips were placed on top of the plastic sheet. Images are representative of three independent experiments.

Figure S3

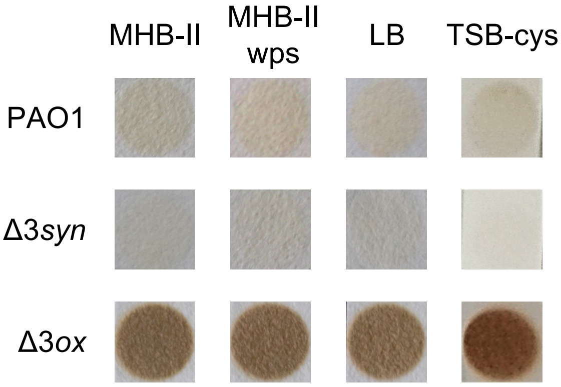

Figure S3. H_2S production under conditions used for MIC assays

Lead acetate-soaked paper strips after reaction with the H_2S released from the PAO1, Δ3*syn*, and Δ3*ox* strains grown for 20 hours in the indicated media under the same experimental conditions used for the MIC assays. Representative data from three independent experiments, each performed on eight bacterial cultures, are shown. MHB-II, Cation-Adjusted Mueller-Hinton Broth; MHB-II wps, MHB-II sealed with plastic sheet; LB, Lysogeny Broth; TSB-cys, Tryptic Soy Broth supplemented with 200 µM L-cysteine.

Figure S4

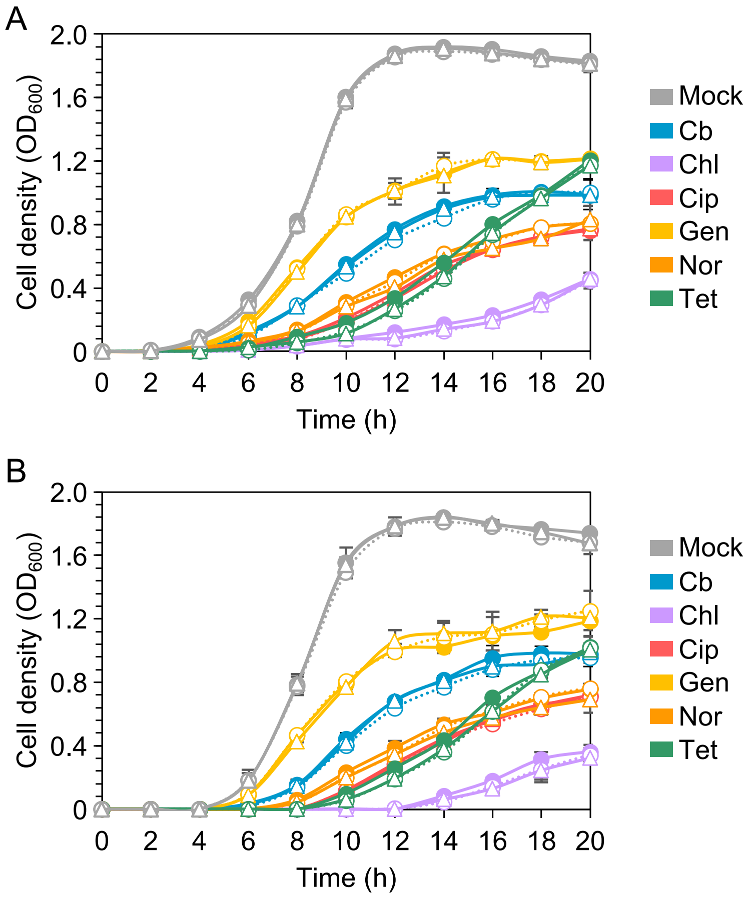

Figure S4. Growth curves in the presence of antibiotics at sub-MIC concentration

Growth curves of PAO1 (solid lines, full circles), Δ3*syn* (dotted lines, empty circles), and Δ3*ox* (solid lines, empty triangles) in LB supplemented with either **(A)** 200 µM L-cysteine or **(B)** 200 µM NaHS, in the absence (mock; gray lines) or in the presence of the following antibiotics at sub-MIC concentrations (1/4 MIC): Cb, carbenicillin (blue lines); Chl, chloramphenicol (purple lines); Cip, ciprofloxacin (red lines); Gen, gentamicin (yellow lines); Nor, norfloxacin (orange lines); Tet, tetracycline (green lines). The average of three independent experiments is reported with standard deviations.

Table S1. Bacterial strains used in this study

Strains	Relevant characteristics	Reference/sources
Escherichia coli		
DH5α	Cloning strain	[S1]
S17.1λ*pir*	Conjugative strain for suicide plasmids	[S2]
Pseudomonas aeruginosa		
PAO1	ATCC 15692 type strain	ATCC
Δ*3mst*	PAO1 derivative strain carrying a deletion of the *3mst* gene (PA1292), obtained by mutagenesis using plasmid pDM4Δ*3mst* (Table S2).	This study
Δ*3syn*	PAO1 Δ*3mst* derivative strain carrying a deletion of the *cbs-cse* gene *locus* (PA0399-PA0400), obtained by mutagenesis using plasmid pDM4Δ*cbs*Δ*cse* (Table S2).	This study
Δ*sqr2*	PAO1 derivative strain carrying a deletion of the *sqr2* gene (PA2345), obtained by mutagenesis using plasmid pDM4Δ*sqr2* (Table S2).	This study
Δ*sqr2*Δ*pdo*	PAO1 Δ*sqr2* derivative strain carrying a deletion of the *pdo* gene (PA2915), obtained by mutagenesis using plasmid pDM4Δ*pdo* (Table S2).	This study
Δ*3ox*	PAO1 Δ*sqr2*Δ*pdo* derivative strain carrying a deletion of the *sqr1* gene (PA2566), obtained by mutagenesis using plasmid pDM4Δ*sqr1* (Table S2).	This study

Table S2. Plasmids used in this study

Plasmids	Relevant characteristics and plasmids construction	Reference/sources
pBluescript II KS(+)	Cloning vector; ColE1 replicon; Amp^R.	Stratagene
pDM4	Suicide vector; *sacBR*; *oriR6K*; Chl^R.	[S3]
pBS-*3mst*UP	The DNA fragment encompassing the upstream region of the *3mst* gene (PA1292), originated with primers FW*3mst*UP and RV*3mst*UP, was cloned in pBluescript II KS(+) by XhoI-EcoRI restriction; Amp^R.	This study
pBS-*3mst*UP-DOWN	The DNA fragment encompassing the downstream region of the *3mst* gene (PA1292), originated with primers FW*3mst*DW and RV*3mst*DW, was cloned in pBS-*3mst*UP by EcoRI-XbaI restriction; Amp^R.	This study
pBS-*cse*DOWN	The DNA fragment encompassing the downstream region of the *cse* gene (PA0400), originated with primers FW*cse*DW and RV*cse*DW, was cloned in pBluescript II KS(+) by NotI-SpeI restriction; Amp^R.	This study
pBS-*cbs*UP-*cse*DOWN	The DNA fragment encompassing the upstream region of the *cbs* gene (PA0399), originated with primers FW*cbs*UP and RV*cbs*UP, was cloned in pBS-*cse*DOWN by SacI-NotI restriction; Amp^R.	This study
pBS-*pdo*UP	The DNA fragment encompassing the upstream region of the *pdo* gene (PA2915), originated with primers FW*pdo*UP and RV*pdo*UP, was cloned in pBluescript II KS(+) by XhoI-EcoRI; Amp^R.	This study
pBS-*pdo*UP-DOWN	The DNA fragment encompassing the downstream region of the *pdo* gene (PA2915), originated with primers FW*pdo*DW and RV*pdo*DW, was cloned in pBS-*pdo*UP by EcoRI-XbaI restriction; Amp^R.	This study
pBS-*sqr2*UP	The DNA fragment encompassing the upstream region of the *sqr2* gene (PA2345), originated with primers FW*sqr2*UP and RV*sqr2*UP, was cloned in pBluescript II KS(+) by XhoI-EcoRI restriction; Amp^R.	This study
pBS-*sqr2*UP-DOWN	The DNA fragment encompassing the downstream region of the *sqr2* gene (PA2345), originated with primers FW*sqr2*DW and RV*sqr2*DW, was cloned in pBS-*sqr2*UP by EcoRI-XbaI restriction; Amp^R.	This study
pBS-*sqr1*UP	The DNA fragment encompassing the upstream region of the *sqr1* gene (PA2566), originated with primers FW*sqr1*UP and RV*sqr1*UP, was cloned in pBluescript II KS(+) by SacI-NotI restriction; Amp^R.	This study
pBS-*sqr1*UP-DOWN	The DNA fragment encompassing the downstream region of the *sqr1* gene (PA2566), originated with primers FW*sqr1*DW and	This study

	RV*sqr1*DW, was cloned in pBS*sqr1*UP by NotI-SpeI restriction; Amp^R.	
pDM4Δ*3mst*	pDM4-derived plasmid used to introduce the *3mst* mutation in *P. aeruginosa* PAO1. The DNA fragments encompassing the upstream and downstream regions of the *3mst* gene were extracted from pBS-*3mst*UP-DOWN and cloned in pDM4 by XhoI-XbaI restriction; Chl^R.	This study
pDM4Δ*cbs*Δ*cse*	pDM4-derived plasmid used to introduce the mutation of the *cbs-cse* gene *locus* in *P. aeruginosa* Δ*3mst*. The DNA fragments encompassing the upstream region of the *cbs* gene and the downstream region of *cse* gene were extracted from pBS-*cbs*UP-*cse*DOWN and cloned in pDM4 by SacI-SpeI restriction; Chl^R.	This study
pDM4Δ*pdo*	pDM4-derived plasmid used to introduce the *pdo* mutation in *P. aeruginosa* PAO1. The DNA fragments encompassing the upstream and the downstream regions of the *pdo* gene were extracted from pBS-*pdo*UP-DOWN and cloned in pDM4 by XhoI-XbaI restriction; Chl^R.	This study
pDM4Δ*sqr2*	pDM4-derived plasmid used to introduce the *sqr2* mutation in *P. aeruginosa* Δ*pdo*. The DNA fragments encompassing the upstream and downstream regions of the *sqr2* gene were extracted from pBS-*sqr2*UP-DOWN and cloned in pDM4 by SacI-SpeI restriction; Chl^R.	This study
pDM4Δ*sqr1*	pDM4-derived plasmid used to introduce the *sqr1* mutation in *P. aeruginosa* Δ*sqr2*Δ*pdo*. The DNA fragments encompassing the upstream and downstream regions of the *sqr1* gene were extracted from pBS-*sqr1*UP-DOWN and cloned in pDM4 by Xho-XbaI restriction; Chl^R.	This study

Table S3. Oligonucleotides used in this study

Name	Sequence (5'→3')[a]	Restriction site
FW*3mst*UP	CCGCTCGAGCGGCATCCCTGGCTGAAG	XhoI
RV*3mst*UP	GGAATTCGGACGACATCGCGGACT	EcoRI
FW*3mst*DW	GGAATTCATCACCGACCCGCGCCG	EcoRI
RV*3mst*DW	GCTCTAGATTGAGCGGCGCCGCGTT	XbaI
FW*cse*DW	ATAAGAATGCGGCCGCAAGATCTGAGGCTCCGCAG	NotI
RV*cse*DW	GGACTAGTAGCCGGGCATCAGCGGC	SpeI
FW*cbs*UP	CGAGCTCCTTCACCTTCGGCCTGTTC	SacI
RV*cbs*UP	ATAAGAATGCGGCCGCGGAAGTCATCTTTTCTCCTTTC	NotI
FW*pdo*UP	CCGCTCGAGGTTGCTGCGACGCCATCC	XhoI
RV*pdo*UP	GGAATTCTTTCAACATGGAGGTTCCTTG	EcoRI
FW*pdo*DW	GGAATTCCCGCCGGTGGAAGGCAAC	EcoRI
RV*pdo*DW	GCTCTAGAGGCGACCACCGCGCCG	XbaI
FW*sqr2*UP	CCGCTCGAGCCGGCTACCTCGTGGGC	XhoI
RV*sqr2*UP	GGAATTCGCTCTGCATGGGAGTTCC	EcoRI
FW*sqr2*DW	GGAATTCACTGGAACGCCATGCTCAAG	EcoRI
RV*sqr2*DW	GCTCTAGACGACCCGCAGCCGGACAT	XbaI
FW*sqr1*UP	CGAGCTCCATGCCGGTACGGCCTTC	SacI
RV*sqr1*UP	ATAAGAATGCGGCCGCTCGTTGCATGACTTCTCCTTA	NotI
FW*sqr1*DW	ATAAGAATGCGGCCGCCCGACCAAGCTTGGCGGG	NotI
RV*sqr1*DW	GACTAGTGATCTCGGAGAAGGTCATGC	SpeI

[a] restriction sites included in the oligonucleotides are underlined.

Detailed Materials and Methods

Bacterial strains and media

Bacterial strains used in this work are listed in **Table S1**. All *E. coli* and *P. aeruginosa* strains were routinely grown at 37°C in Lysogeny Broth (LB), in Cation-adjusted Mueller-Hinton Broth (MHB-II), or in Tryptic Soy Broth (TSB), in static or shaking conditions (200 rpm), or in LB supplemented with 15 g/L agar. When required, media were supplemented with 200 µM L-cysteine or 200 µM NaHS (these concentrations of L-cysteine and NaHS were selected based on previous studies [S4, S5]). Unless otherwise stated, antibiotics were added at the following concentrations: ampicillin (Amp) 100 µg/mL (*E. coli*); nalidixic acid (Nal) 15 µg/mL (*E. coli*); chloramphenicol (Chl) 30 µg/mL (*E. coli*) or 375 µg/mL (*P. aeruginosa*).

Recombinant DNA techniques

Plasmids and oligonucleotides used in this study are listed in **Table S2** and **Table S3**, respectively. Plasmid DNA extraction, purification of DNA fragments, restriction enzyme digestions, ligations, and transformations in *E. coli* DH5α or S17.1λ*pir* competent cells were performed by using standard protocols [S6]. DNA amplification *via* PCR was performed using the GoTaq Polymerase (Promega, Madison, WI). FastDigest restriction enzymes were purchased from Thermo Fisher Scientific (Waltham, MA). DNA fragments ligation was performed using T4 DNA Ligase (Promega). Plasmids were introduced into *P. aeruginosa* by transformation or bi-parental conjugation using *E. coli* S17.1λ*pir* as the donor strain [S6]. Restriction analysis and DNA sequencing were performed to check the plasmids generated in this work (details on plasmids construction are reported in **Table S2**).

Construction of deletion mutants

P. aeruginosa mutant strains were generated by allelic exchange using pDM4-derivative plasmids, as previously described [S3,S7]. Details on pDM4-derivative plasmids construction are provided in **Table S2**. Plasmids were independently introduced into *P. aeruginosa* strains following conjugal mating with *E. coli* S17.1λ*pir* as the donor strain [S6]. Clones with a chromosomal insertion of the pDM4-derivative plasmids were selected on LB agar plates supplemented with 375 µg/mL Chl and 15 µg/mL Nal. Plasmid excision from the chromosome was subsequently selected on LB agar plates supplemented with 10% (w/v) sucrose. Resulting mutant strains were confirmed by PCR analysis.

H_2S detection

To monitor H_2S released by PAO1, its isogenic mutants or *P. aeruginosa* clinical isolates from cystic fibrosis patients, lead acetate-soaked paper strips were used as previously described [S4,S5,S8]. Paper strips saturated with 2% $Pb(Ac)_2$ were affixed over the wells of a 96-well microtiter plate, in the gas phase above the liquid cultures (100 µL). When indicated, an adhesive plastic sheet (AriaMx Adhesive Plate Seals, Agilent) not permeable to H_2S (**Figure S2**) was used to seal the wells and limit possible H_2S leakage. Overnight cultures were diluted 1:100 in LB, MHB-II or TSB supplemented with 200 µM L-cysteine (TSB-cys), and incubated for 20 hours at 37°C in static conditions. After incubation, paper strips were gently removed, scanned, and analysed by densitometry with ImageJ. Relative H_2S release was determined from the densitometric value obtained for each culture after correction for the background value (*i.e.* the densitometric value obtained for the non-inoculated medium) and normalization to the OD_{600} of the corresponding culture. Relative H_2S level were determined in three independent experiments.

MIC assays

The Minimal Inhibitory Concentration (MIC) of the antibiotics carbenicillin (Cb), chloramphenicol (Chl), ciprofloxacin (Cip), colistin (Col), gentamicin (Gen), meropenem (Mer), norfloxacin (Nor), tetracycline (Tet), and tobramycin (Tob) was determined by the standard broth microdilution method in MHB-II, LB and TSB-cys according to the Clinical and Laboratory Standards Institute guidelines [S9], or with minor modifications of the standard protocol. Briefly, *P. aeruginosa* PAO1 and its isogenic mutants were grown at 37°C with shaking in MHB-II, LB, or TSB. After 16 hours, cultures were diluted in 96-well microtiter plates to an OD_{600} of ≈ 0.0005 (ca. 5×10^5 CFU/mL) in 100 µL of MHB-II, LB or TSB-cys, supplemented with increasing concentrations of the antibiotics indicated in the main text. Since H_2S is a volatile molecule, the PAO1, Δ3*syn*, and Δ3*ox* strains were grown in distinct 96-well microtiter plates, and adhesive plastic sheets (AriaMx Adhesive Plate Seals, Agilent) were used to seal the wells when specified in the text (wps). MIC was visually defined as the lowest concentration of antibiotic able to inhibit bacterial growth after 20 hours of incubation at 37°C under static conditions. All strains were tested in all conditions in at least three independent experiments.

Generation of growth curves

Growth curves of *P. aeruginosa* PAO1 and the isogenic Δ3*syn* and Δ3*ox* mutants were generated as follows. Bacterial cultures were grown at 37°C with shaking in LB. After 16 hours, cultures were

diluted in 96-well microtiter plates to an OD_{600} of ≈ 0.0005 (ca. 5 × 10^5 CFU/mL) in 100 µL of LB (supplemented or not with 200 µM L-cysteine or 200 µM NaHS). Antibiotics (*i.e.*, Cb, Chl, Cip, Gen, Nor, or Tet) were added at concentrations corresponding to 1/4 of the MIC value obtained in LB (refer to **Table 1** in the main text). As detailed for MIC assays, the PAO1, Δ3*syn*, and Δ3*ox* strains were grown in distinct 96-well microtiter plates to avoid possible effects of H_2S produced by the PAO1 and Δ3*ox* strains on the Δ3*syn* strain; comparable results were obtained when the PAO1, Δ3*syn*, and Δ3*ox* strains were grown in the same microtiter plate. OD_{600} values were recorded every hour for 20 hours using an automated luminometer-spectrophotometer plate reader Spark10M (Tecan). All strains were tested in all conditions in at least three independent experiments. These experiments were performed with and without an adhesive plastic sheet (AriaMx Adhesive Plate Seals, Agilent) used to seal the wells, yielding comparable results.

Statistical analyses

Statistical analyses were performed using the GraphPad Prism 6.01 software (https://www.graphpad.com/). Unpaired *t*-test (single comparison) was used for comparisons between two groups. Differences in data distributions of H_2S production in *P. aeruginosa* clinical strains were statistically tested using the non-parametric Kolmogorov-Smirnov test. In all cases, *P* values lower than 0.05 were considered statistically significant.

References

S1. Grant SG, Jessee J, Bloom FR, Hanahan D. 1990. Differential plasmid rescue from transgenic mouse DNAs into *Escherichia coli* methylation-restriction mutants. Proc Natl Acad Sci USA 87:4645-4649.

S2. Simon R, Priefer U, Pühler A. 1983. A broad host range mobilization system for *in vivo* genetic engineering: transposon mutagenesis in Gram-negative bacteria. Nat Biotechnol 1:784-791.

S3. Milton DL, O'Toole R, Horstedt P, Wolf-Watz H. 1996. Flagellin A is essential for the virulence of *Vibrio anguillarum*. J Bacteriol 178:1310-1319.

S4. Shatalin K, Shatalina E, Mironov A, Nudler E. 2011. H_2S: a universal defense against antibiotics in bacteria. Science 334:986-890.

S5. Shatalin K, Nuthanakanti A, Kaushik A, Shishov D, Peselis A, Shamovsky I, Pani B, Lechpammer M, Vasilyev N, Shatalina E, Rebatchouk D, Mironov A, Fedichev P, Serganov A, Nudler E. 2021. Inhibitors of bacterial H_2S biogenesis targeting antibiotic resistance and tolerance. Science 372:1169-1175.

S6. Sambrook J, Fritsch EF, Maniatis T. 1989. Molecular cloning: a laboratory manual, 2nd ed. Cold Spring Harbor Laboratory Press, Cold Spring Harbor, New York.

S7. Letizia M, Mellini M, Fortuna A, Visca P, Imperi F, Leoni L, Rampioni G. 2022. PqsE expands and differentially modulates the RhlR quorum sensing regulon in *Pseudomonas aeruginosa*. Microbiol Spectr 10:e0096122.

S8. Forbes BA, Sahm DF, Weissfeld AS. 1998. Bailey and Scott's Diagnostic Microbiology. Mosby Inc, 10th ed.

S9. Clinical and Laboratory Standards Institute. 2012. Methods for dilution antimicrobial susceptibility tests for bacteria that grow aerobically. Wayne PA: CLSI (Approved standard M07-A9), 9th ed.
